# Effect of different carbon dioxide (CO_2_) insufflation for laparoscopic colorectal surgery in elderly patients: A randomized controlled trial

**DOI:** 10.1097/MD.0000000000017520

**Published:** 2019-10-11

**Authors:** Rongjuan Jiang, Yan Sun, Huaiming Wang, Min Liang, Xianfeng Xie

**Affiliations:** a Department of Anesthesiology, Chengdu Second People's Hospital; b Department of Anesthesiology, Sichuan Cancer Hospital, Chengdu, Sichuan; c Department of Anesthesiology, Liaocheng People's Hospital, Liaocheng, Shandong, China.

**Keywords:** carbon dioxide, elderly patients, hypothermia, laparoscopic colorectal surgery, postoperative pain

## Abstract

**Background:**

Evidence suggests that dry CO_2_ insufflation during laparoscopic colorectal surgery results in greater structural injury to the peritoneum and longer hospital stay than the use of warm, humidified CO_2_. We aimed to test the hypothesis that warm, humidified CO_2_ insufflation could reduce postoperative pain and improve recovery in laparoscopic colorectal surgery.

**Methods:**

One hundred fifty elderly patients undergoing laparoscopic colorectal surgery under general anesthesia from May 2017 to October 2018 were randomly divided into 3 groups. The primary outcomes were resting pain, cough pain, and consumption of sufentanil at 2, 4, 6, 12, 24, and 48 hours postoperatively. Quality of visual image, hemodynamic changes, esophageal temperature, mean skin temperature, mean body temperature, recovery time, days to first flatus and solid food intake, shivering, incidence of postoperative ileus, length of hospital stay, surgical site infections, patients and surgeon satisfaction scores, adverse events, prothrombin time, activated partial thromboplastin time, and thrombin time were recorded.

**Results:**

Group CE patients were associated with significantly higher early postoperative cough pain and sufentanil consumption than the other 2 groups (*P* < .05). Compared with group CE, patients in both groups WH and CF had significantly reduced intraoperative hypothermia, recovery time of PACU, days to first flatus and solid food intake, and length of hospital stay, while the satisfaction scores of both patients and surgeon were significantly higher (*P* < .05). Prothrombin time, activated partial thromboplastin time, and thrombin time were significantly higher in group CE from 60 minutes after pneumoperitoneum to the end of pneumoperitoneum than the other 2 groups (*P* < .05). The number of patients with a shivering grade of 0 was significantly lower and grade of 3 was significantly higher in group CE than in the other 2 groups (*P* < .05).

**Conclusion:**

Use of either warm, humidified CO_2_ insufflations or 20°C, 0% relative humidity CO_2_ combined with forced-air warmer set to 38°C during insufflations can both reduce intraoperative hypothermia, dysfunction of coagulation, early postoperative cough pain, sufentanil consumption, days to first flatus, solid food intake, and length of hospital stay.

## 1 Introduction

Gastrointestinal tumors are one of the most common causes of cancer-related death worldwide over the last decade. Surgical resection is the main mode of treatment.^[1,2]^ With rapid global increase in the aging population, laparoscopic surgery is a popular option to reduce operative morbidity, for the advantages it offers, such as minimized incision, less postoperative pain and blood loss, shorter hospital stay, and faster recovery than traditional surgical options.^[3]^ However, laparoscopic procedures also have some disadvantages, namely prolonged operation time and impact of carbon dioxide (CO_2_) pneumoperitoneum on the respiratory and circulatory system.^[4]^

CO_2_ is still the most commonly used gas during laparoscopic surgery because it is noncombustible, cost effective, and easily excretable from the respiratory system, although it has different biophysical properties under different thermal conditions.^[5]^ Long-term laparoscopic surgery requires prolonged CO_2_ absorption and accumulation, eventually causing hypercapnia, which may lead to hypertension, tachycardia, and other complications.^[6]^ Previous studies have also reported that the injury and detrimental effects of CO_2_ insufflation during laparoscopic surgery may be due to both physical and chemical factors such as hypothermia and cellular acidification.^[7,8]^

Maintaining normothermia during operation is strongly recommended by the enhanced recovery after surgery (ERAS) guidelines, as even a mild degree of intraoperative hypothermia (34.0–36°C) could dramatically increase surgical complications and morbidity.^[9]^ General anesthesia and insufflation of gas at ambient temperatures during laparoscopic abdominal surgery maybe associated with impaired thermoregulation due to prolonged procedure times.^[10]^ Despite multiple available strategies being used, inadvertent hypothermia is common in laparoscopic colorectal surgery. As a result, intraoperative warming should be considered as part of the anesthetic management when patients are at risk of hypothermia.^[11]^ Forced-air warming devices have been reported to be one of the most effective methods to achieve normothermia during abdominal surgery. A previous study found than even 30 minutes of forced-air warming before the induction of anesthesia could increase peripheral tissue heat content and reduce intraoperative hypothermia.^[12]^ Another study confirmed that warm and humidified gas insufflation during laparoscopic surgery is an alternative active method to prevent hypothermia.^[13]^ However, the use of a device to heat and humidify the gas may actually add to the cost and complexity of the procedure.^[14]^ We designed this trial to determine the effect of warm and humidified gas insufflation compared to cold gas insufflation with forced-air warming devices or electric blankets in maintaining intraoperative normothermia for elderly patients undergoing laparoscopic colorectal surgery.

## 2 Materials and methods

### 2.1 Patients

We recruited 150 elderly patients undergoing laparoscopic colorectal surgery under general anesthesia from May 2017 to October 2018. All patients were managed according to the ERAS guidelines that included preoperative oral carbohydrate drinks; minimization of perioperative intravenous (IV) fluids; bowel preparation; early introduction of postoperative oral diet; early patient mobilization; and omission or early removal of drains, lines, nasogastric tubes, and urinary catheters.^[15]^

All patients provided informed written consent, and ethical approval was obtained from both the Institutional Review Board of Liaocheng People's Hospital and Chengdu Second People's Hospital, China. This trial was also registered at chictr.org (ChiCTR-IOR-17010915). Patients were randomized into the following 3 groups using random numbers based on a random number generator: group WH (received warm [37°C], humidified [98% relative humidity] CO_2_ insufflation, n = 50); group CE (received 20°C, 0% relative humidity CO_2_ insufflation, intraoperative warming with electric blankets set to 38°C, n = 50); and group CF (received 20°C, 0% relative humidity CO_2_ insufflation, intraoperative warming with forced-air warmer set to 38°C, n = 50). The criteria for inclusion were age between 65 and 75 years, American Society of Anesthesiology (ASA) Grades I to III, confirmed diagnosis of colorectal carcinoma by pathology and no metastases by computed tomography (CT), and scheduled for laparoscopic colorectal resection under general anesthesia. The criteria for exclusion were a history of alcohol or drug abuse, age more than 75 years and less than 65 years, evidence of current infection, thyroid disease, use of cannabinoids or corticosteroids, and body mass index (BMI) >30 kg/m^2^.

### 2.2 Operative procedure

The room temperature was maintained at 22 to 24°C and relative humidity at 40% to 60%. On arrival in the operating room, routine anesthetic monitors were attached. After Modified Allen's test was carried out to ensure patency of collateral circulation of the hand, a 20 G catheter was inserted into the radial artery for continuous arterial pressure monitoring and blood sampling. Anesthesia was induced with propofol, fentanyl, and cisatracurium. Next, a transesophageal temperature probe with an accuracy of 0.1°C was inserted via the nasal route and fixed in position at the estimated junction between the middle and lower third of the esophagus according to a previous study.^[16]^ End-tidal pressure of carbon dioxide (PetCO_2_) was maintained between 35 and 45 mm H_2_O by adjusting minute ventilation and respiratory frequency for patients undergoing volume controlled ventilation after pneumoperitoneum. Pneumoperitoneum was established with CO_2_ gas using a Stryker 40L High flow Insufflator (Stryker Endoscopy, San Jose, CA, USA). Intraabdominal pressure was maintained 12 mm Hg, and the upper gas flow limit was 6.5 L/minute. Patients in both groups CE and CF received 20°C, 0% relative humidity CO_2_ insufflation; the only difference between the 2 groups was that patients in group CE were warmed with electric blankets set to 38°C, while those in group CF were warmed with forced-air warmer set to 38°C (Bair Hugger; Augustine Medical, Eden Prairie, MN, USA). The CO_2_ conditioning device delivered warmed (37°C) and humidified (98%) CO_2_ to the patients in group WH via a dual-lumen insulated tubing system. Intravenous fluids were infused intraoperatively using an Animec warmer (Elltec Co. Ltd., Nagoya, Japan) during surgery. All laparoscopic procedures were performed by the same experienced laparoscopic surgery team. When systolic blood pressure exceeded ±20% of the baseline value, we administered phenylephrine (40 μg), ephedrine (6 mg), or urapidil (10 mg) at 5-minute intervals. Atropine (0.4 mg) was administered at 5-minute intervals when the heart rate fell to 80% below the baseline value or was lower than 50 beats/minute.

After surgery, all patients were managed with IV patient-controlled analgesia (PCA) using sufentanil with the following settings: 1.6 μg per bolus, bolus lock for 5 minutes, and a maximum of 6.4 μg/hour. Ketorolac or hydromorphone was given if visual analogue scale (VAS) score of rest pain was ≥4. The mean skin temperature (Tskin) was calculated as following: Tskin = 0.3 (Tchest + Tarm) + 0.2 (Tthigh + Tcalf). The mean body temperature (MBT) was estimated from the core and mean skin temperatures according to a previous study. Briefly, MBT = 0.64 × Tcore + 0.36 × Tskin.^[12]^ In the post-anesthesia care unit (PACU), infrared tympanic thermometers were used to measure core temperatures, and hypothermic patients received only forced-air warming at the discretion of anesthesiologists. Deep vein thrombosis prophylaxis was performed with low molecular weight heparin (50 IU/kg/d) after surgery.^[17]^

### 2.3 Outcome measures

Postoperative analgesia was administered by nursing staff in the acute pain service (APS) who were blinded to this trial. The primary outcome measures were resting pain, cough pain, and consumption of sufentanil at 2, 4, 6, 12, 24, and 48 hours postoperatively. The quality of visual image (on a Likert scale from 1 to 10 [1 = perfect image, 10 = very poor quality image])^[18]^; hemodynamic changes (mean arterial pressure [MAP]) and heart rate (HR) were recorded at the following time points: arrival in the operation room (T0), just before the induction of anesthesia (T1), at the beginning of pneumoperitoneum (T2), 10 minutes (T3), 20 minutes (T4), 30 minutes (T5), and 60 minutes (T6) after the pneumoperitoneum, at the end of operation (T7), and 5 minutes (T8), 10 minutes (T9), and 15 minutes (T10) after arriving at the PACU; esophageal temperature; mean skin temperature; mean body temperature; recovery time; days to first flatus and solid food intake; shivering (using a scale validated by Crossley and Mahajan: 0 = no shivering, 1 = piloerection or peripheral vasoconstriction but no visible shivering, 2 = muscular activity in only 1 muscle group, 3 = muscular activity in more than 1 muscle group but not generalized, and 4 = shivering involving the whole body)^[19]^; incidence of postoperative ileus (POI, defined as the absence of flatus and/or passage of stool or intolerance of oral intake after postoperative day 3, with radiographic confirmation of small and/or large intestinal dilatation on an abdominal radiograph according to a previous study)^[20]^; discharge time; surgical site infections (SSIs, defined using both objective clinical and microbiological criteria according to guidance from Public Health England, were recorded within 14 postoperative days)^[21]^; patients and surgeon satisfaction scores (on a 5-point scale: 5 = excellent, 4 = adequate, 3 = cannot say, 2 = inadequate, and 1 = poor)^[22]^; and adverse events were also recorded. Prothrombin time, activated partial thromboplastin time, and thrombin time were measured on the day before surgery, start of pneumoperitoneum, 60 minutes after pneumoperitoneum, and at the end of pneumoperitoneum.

### 2.4 Statistical analysis

The sample size was calculated on the basis of an expected 20% reduction in the consumption of sufentanil at 48 hours postoperatively. For a study power of 80% (α = .05, β = .2), the required sample size per group was calculated to be 41 (PASS 11.0, NCSS Statistical Software, Kaysville, Utah). Assuming a dropout rate of 20%, the final sample size was determined to be 50 patients for each group.

The Kolmogorov–Smirnov test was used to assess the distribution of variables. Homogeneity of variance was determined using Levene test. Quantitative data were expressed as means and standard deviations, or medians and inter-quartile ranges (IQRs). Inter-group comparisons were performed using repeated-measures analysis of variance (ANOVA). Bonferroni correction was used for post-hoc multiple comparisons. The non-parametric Wilcoxon–Mann–Whitney test was used for variables that were non-normally distributed. Categorical data were expressed as frequencies and percentages, and analyzed using Chi-Squared tests or Fisher exact tests, when appropriate. Probability (*P*) values <.05 were considered statistically significant. All statistical analysis was performed with SPSS for Windows version 22.0 (SPSS Inc., Chicago, IL, USA).

## 3 Results

### 3.1 Patient demographic and operative data

**Figure 1:**
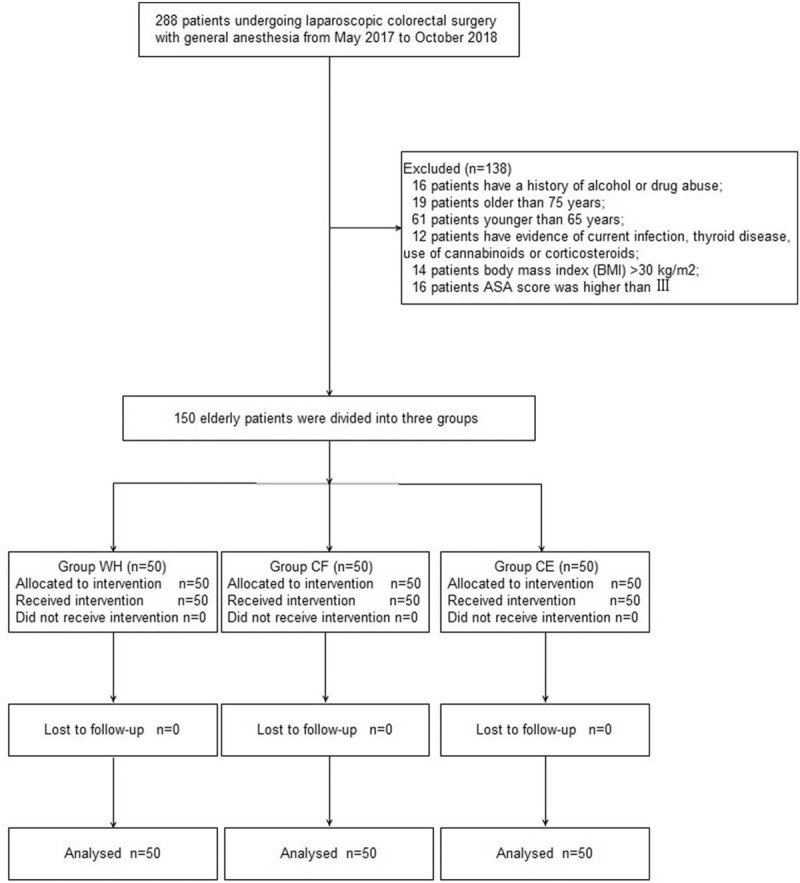
Flow diagram depicting patient enrollment.

Figure 1 depicts the flow chart of patient enrollment. We recruited 288 patients undergoing laparoscopic colorectal surgery under general anesthesia from May 2017 to October 2018. In all, 138 patients were excluded for the following reasons: 16 patients had a history of alcohol or drug abuse; 19 patients were older than 75 years; 61 patients were younger than 65 years; 12 patients showed evidence of current infection, thyroid disease, and use of cannabinoids or corticosteroids; 14 patients had BMI >30 kg/m^2^; and 16 patients’ ASA scores were higher than III. Finally, 150 patients were enrolled in this trial.

There were no significant differences among the 3 groups with respect to age, BMI, gender, ASA grade, operation room (OR) temperature, comorbidity, blood loss, time of anesthesia and operation, surgical procedures, conversion to laparotomy, and volume of CO_2_ (Table 1).

**Table 1:**
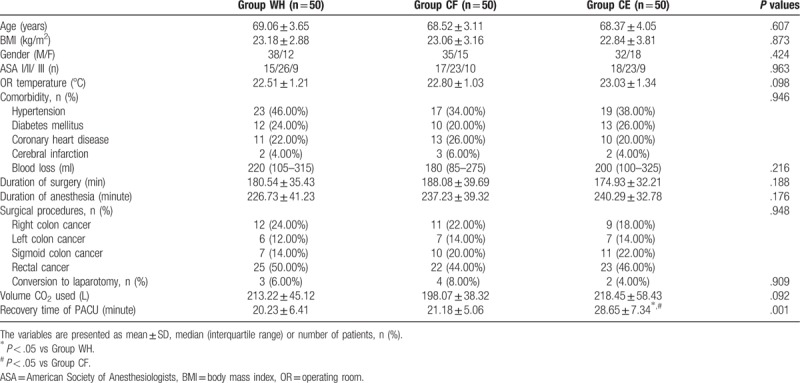
Patients’ characteristics and intraoperative data.

### 3.2 Intraoperative variables

**Figure 2:**
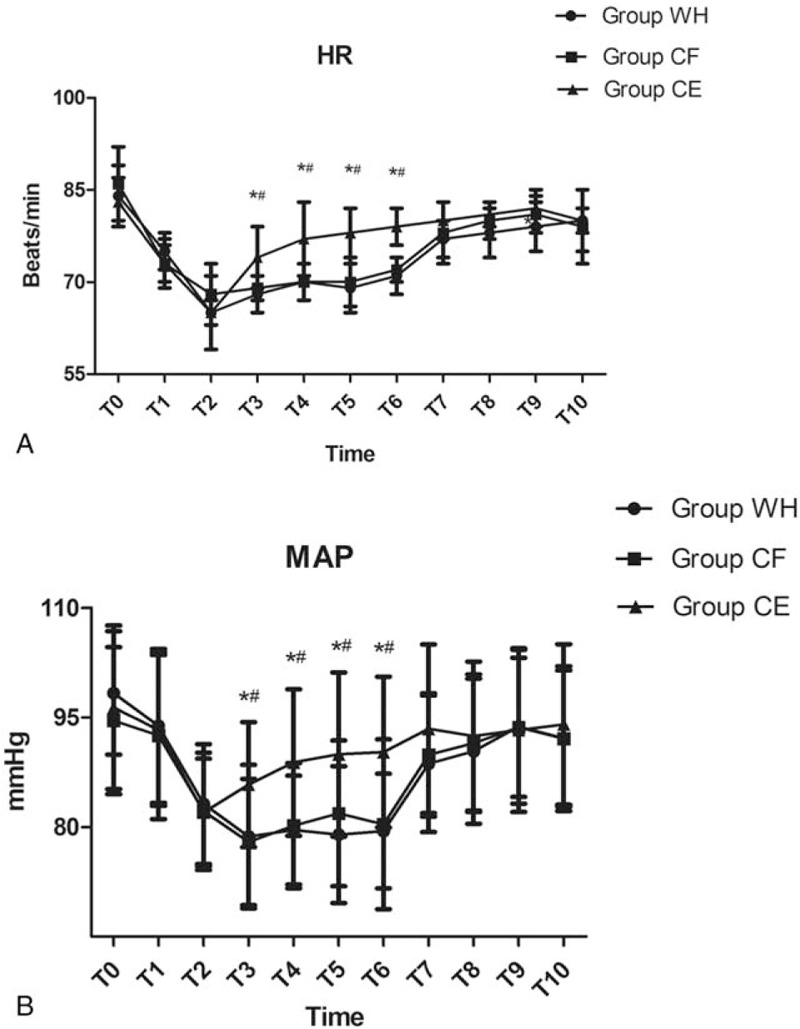
Hemodynamic data (HR and MAP) for the 3 groups of patients at the following timepoints: arrival in the operation room (T0), just before the induction of anesthesia (T1), at the beginning of pneumoperitoneum (T2), 10 minutes (T3), 20 minutes (T4), 30 minutes (T5), 60 minutes (T6) after the pneumoperitoneum, at the end of operation (T7), and 5 minutes (T8), 10 minutes (T9), and 15 minutes (T10) after arriving at the PACU. ∗*P* < .05 vs Group WH, ^#^*P* < .05 vs Group CF.

Both HR and MAP were not significantly different among the 3 groups at the time of arrival in the operation room (*P* > .05, Fig. 2A and B). Compared with groups WH and CF, both HR and MAP in group CE were significantly higher from T3 to T6 (*P* < .05, Fig. 2A and B). There was no significant difference between group WH and CF with respect to both HR and MAP from T0 to T10 (*P* > .05, Fig. 2A and B).

**Figure 3:**
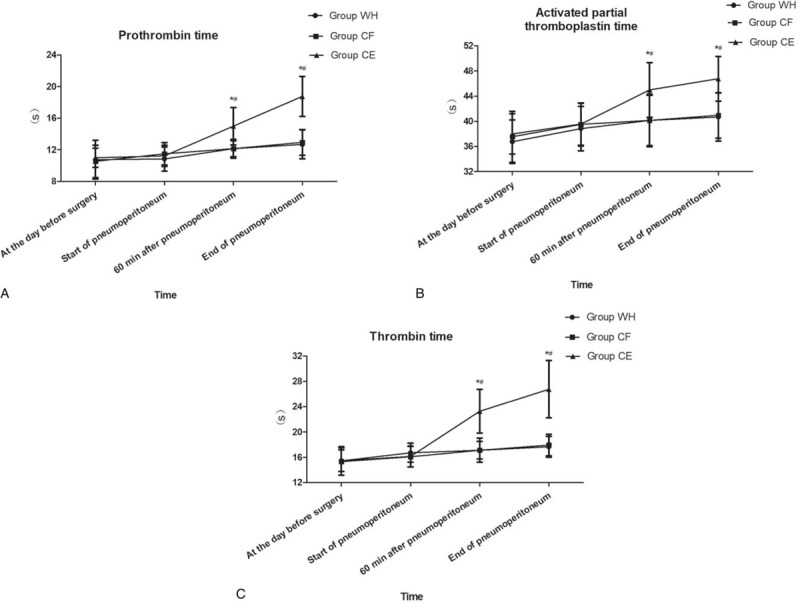
Prothrombin time, activated partial thromboplastin time, and thrombin time for the 3 groups of patients at the following timepoints: at the day before surgery, start of pneumoperitoneum, 60 minutes after pneumoperitoneum, and the end of pneumoperitoneum. ∗*P* < .05 vs Group WH, ^#^*P* < .05 vs Group CF.

The prothrombin time, activated partial thromboplastin time, and thrombin time in groups WH and CF did not have significant alterations from the day before surgery to the end of pneumoperitoneum (*P* > .05, Fig. 3A–C). However, compared with groups WH and CF, prothrombin time, activated partial thromboplastin time, and thrombin time in group CE were significantly higher from 60 minutes after pneumoperitoneum to the end of pneumoperitoneum (*P* < .05, Fig. 3A–C).

**Figure 4:**
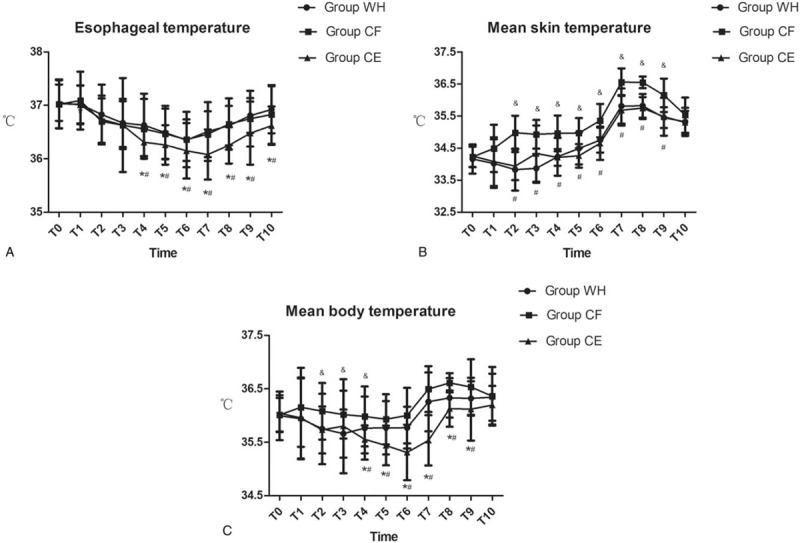
Esophageal temperature, mean skin temperature, and mean body temperature for the 3 groups of patients at the following timepoints: arrival in the operation room (T0), just before the induction of anesthesia (T1), at the beginning of pneumoperitoneum (T2), 10 minutes (T3), 20 minutes (T4), 30 minutes (T5), 60 minutes (T6) after the pneumoperitoneum, at the end of operation (T7), and 5 minutes (T8), 10 minutes (T9), and 15 minutes (T10) after arriving at the PACU. ∗*P* < .05 vs Group WH, ^#^*P* < .05 vs Group CF, ^&^*P* < .05 Group CF vs Group WH.

The esophageal temperature in group CE was significantly lower than in groups WH and CF from T4 to T10 (*P* < .05, Fig. 4A). There was no significant difference between groups WH and CF with respect to esophageal temperatures from T0 to T10 (*P* > .05, Fig. 4A). However, compared with groups WH and CE, the mean skin temperature in group CF was significantly higher from T2 to T9 (*P* < .05, Fig. 4B). There was no significant difference between groups WH and CE with respect to mean skin temperature from T0 to T10 (*P* > .05, Fig. 4B). The mean body temperature in group CE was significantly lower from T4 to T9 than that in groups WH and CF (*P* < .05, Fig. 4C). At the same time, mean body temperature in group CF was significantly higher from T2 to T4 than group WH (*P* < .05, Fig. 4C).

The recovery time of PACU was significantly longer in group CE than in groups WH and CF (*P* < .05, Table 1); whereas, there was no significant difference between groups WH and CF with respect to the recovery time of PACU (*P* < .05, Table 1). The satisfaction scores of both patients and surgeon were significantly higher in groups WH and CF than group CE (*P* < .05, Table 2).

**Table 2:**
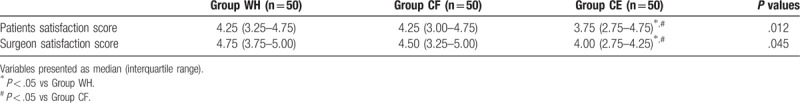
Satisfaction scores of patients and surgeons.

### 3.3 Postoperative variables

**Figure 5:**
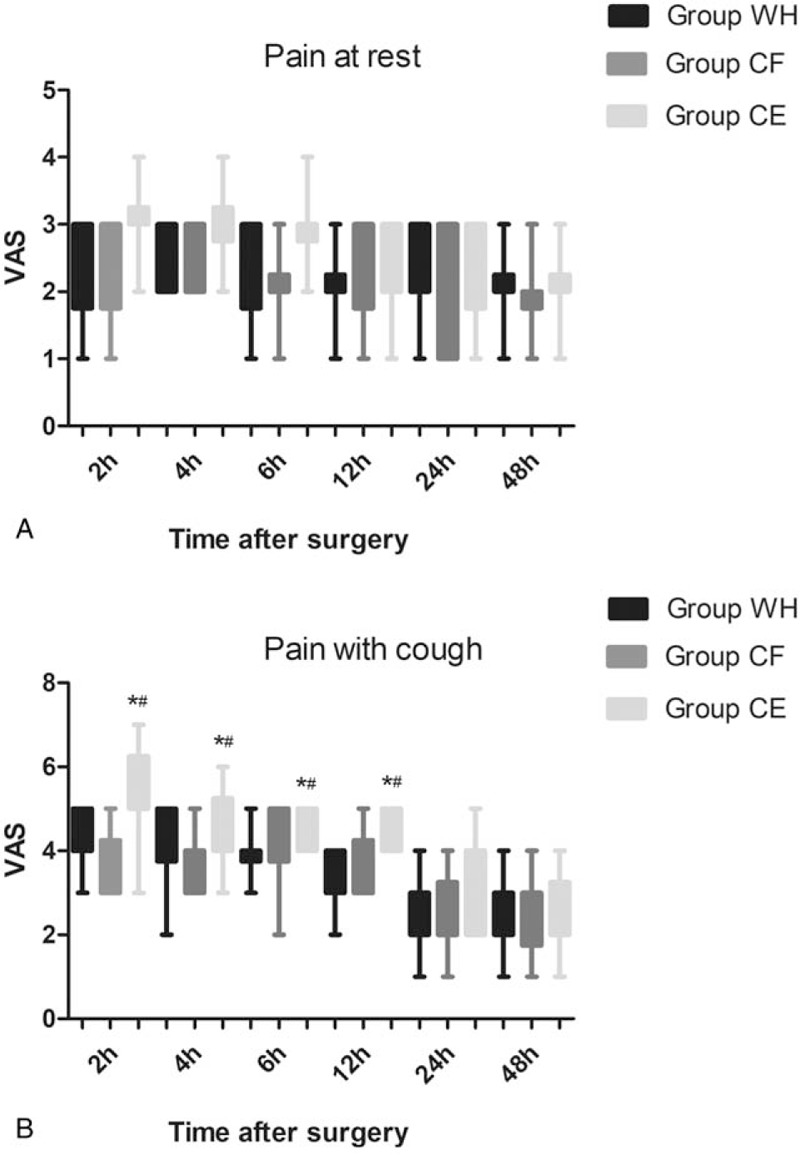
Pain scores (both at rest and with cough) at 2, 4, 6, 12, 24, and 48 hours postoperatively for the 3 groups of patients. ∗*P* < .05 vs Group WH, ^#^*P* < .05 vs Group CF.

**Figure 6:**
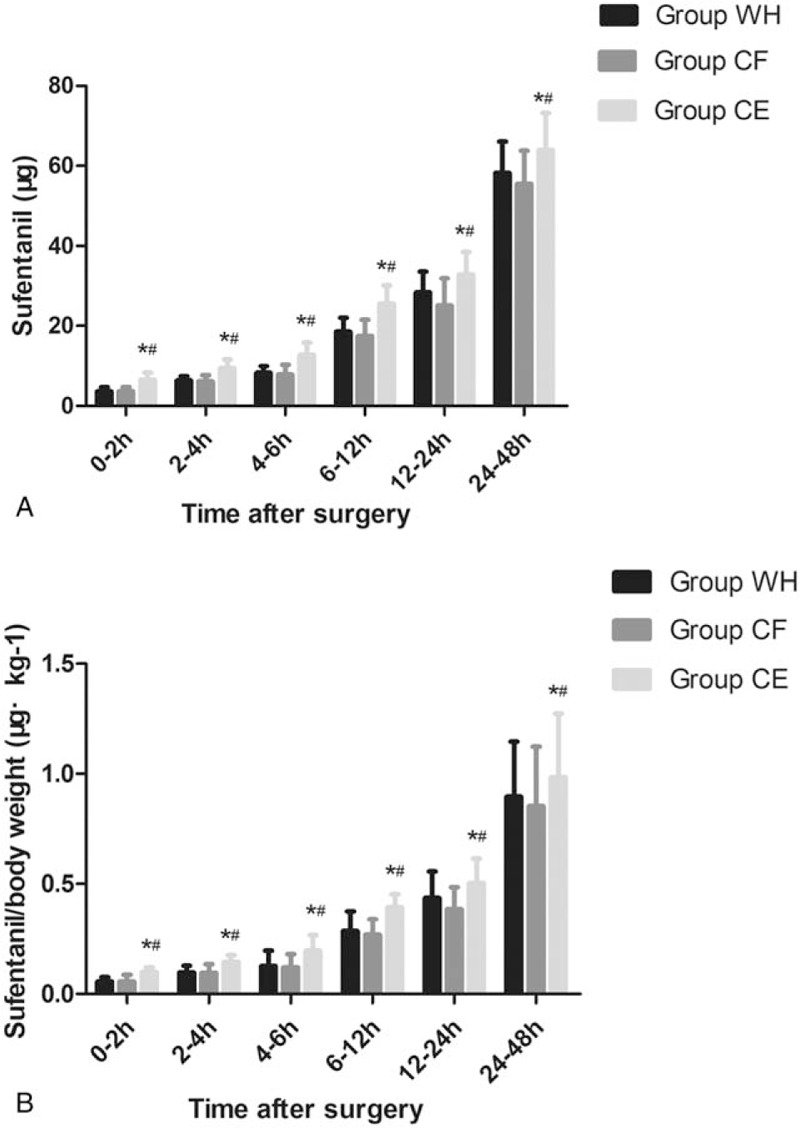
Consumption of sufentanil (both total dosage and dosage per body weight) at 2, 4, 6, 12, 24, and 48 hours postoperatively for the 3 groups of patients. ∗*P* < .05 vs Group WH, ^#^*P* < .05 vs Group CF.

There was no significant difference among the 3 groups with respect to pain at rest (*P* > .05, Fig. 5A). The VAS scores with cough at 2, 4, 6, and 12 hours after surgery were significantly higher in group CE than in groups WH and CF (*P* < .05, Fig. 5B). However, there was not significantly different between group WH and CF with the respect to pain with cough (*P* > 0.05, Fig. 5B). The total dosage and dosage per body weight of sufentanil were significantly higher in groups WH and CF than in group CE at 2, 4, 6, 12, 24, and 48 hours after surgery (*P* < .05, Fig. 6A and B).

There was no significant difference among the 3 groups with respect to the quality of visual image, occurrence of vomiting, POI, and SSI (*P* > .05, Table 3). However, the discharge time, time to first flatus, and solid food intake were significantly higher in group CE than groups WH and CF (*P* < .05, Table 3).

**Table 3:**
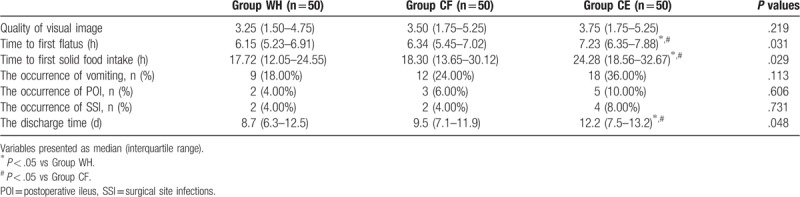
Postoperative clinical parameters among the 3 groups.

There was no significant difference among the 3 groups with respect to adverse events such as arrhythmia, hypertension, and hypotension except delirium. Although the number of patients with a shivering grade of 0 was significantly lower and a grade of 3 was significantly higher in group CE than in the other 2 groups, there was no significant difference among the 3 groups with respect to shivering (*P* > .05, Table 4).

**Table 4:**
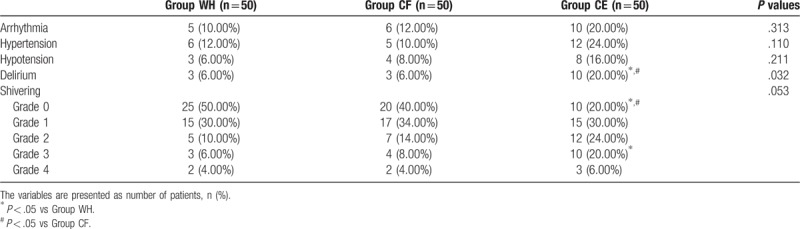
Comparison of the adverse events among the 3 groups.

## 4 Discussion

Our study revealed that patients with warm, humidified CO_2_ or 20°C, 0% relative humidity CO_2_ combined with forced-air warmer set to 38°C during insufflations could significantly reduce intraoperative hypothermia, coagulation dysfunction, recovery time of PACU, days to first flatus and solid food intake, early postoperative cough pain, sufentanil consumption, and length of postoperative hospital stay than patients with 20°C, 0% relative humidity CO_2_ combined with electric blankets set to 38°C during insufflation.

The concept of enhanced recovery, which provides patients with optimal ways to minimize the deleterious effects of surgery, has been widely used in gastrointestinal surgery.^[23]^ According to guidelines in French, the fast-track programs of colorectal surgery usually include the following aspects: surgical stress, fluid and electrolyte imbalances, postoperative ileus, decreased postoperative mobility, sleep disorders, and postoperative complications.^[24]^ A previous study also found that the complication rate reduced to 20% and associated mortality was 3.4% after laparoscopic colorectal surgery.^[25]^ H, all patients undergoing laparoscopic colorectal surgery had been managed as per the ERAS guidelines in our hospital since 2015.

We did not find any significant differences in pain at rest during the first 48 hours after surgery among the 3 groups. However, both pain with cough during 12 hours after surgery and postoperative sufentanil consumption during 48 hours after surgery were higher in group CE than the other 2 groups. Many factors can influence the postoperative pain after laparoscopic colorectal resection, such as abdominal incisions, pneumoperitoneum, operative dissection, and secondary peritonitis and ileus.^[26,27]^ Previous studies have found that cold-dry CO_2_ insufflation could desiccate the peritoneum, damage the peritoneal surface, induce inflammatory response, and then release various hyperalgesic substances such as kinins and prostaglandins.^[28,29]^ Other studies found that prolonged cold-dry CO_2_ insufflation during laparoscopic colorectal surgery could also result in serious peritoneal injury, which may increase the risk of peritoneal metastasis and port site metastasis.^[29–31]^ Patients are prone to sputum retention, atelectasis, and pneumonia if postoperative pain is poorly controlled although a previous study did not find any difference with respect to the incidence of postoperative respiratory complication because of the smaller sample size.^[32]^ Whether insufflating warmed, humidified CO_2_ is clinically advantageous for patients undergoing laparoscopic colorectal surgery remains to be elucidated. Besides, it is generally accepted that the pressure of CO_2_ used for the pneumoperitoneum may also contribute to peritoneal injury.^[33]^ As a result, we adopted the lower pressure pneumoperitoneum than previous studies in this trial.

In our trial, patients were considered to be at higher risk of perioperative hypothermia, because they had the following characteristics: preoperative mean body temperature below 36.0°C, undergoing major surgery, and elderly patients with risk of cardiovascular complications. Although previous studies have found that intraoperative mild hypothermia can reduce the metabolic rate and oxygen consumption and increase tolerance of tissues and organs to ischemia and hypoxia, they have also reported that long-term perioperative hypothermia can result in peripheral vasoconstriction, impairment of coagulation capability and myocardial contractility, increased perioperative oxygen consumption, and SSIs.^[34–36]^ A previous study also reported that intraoperative normothermia could reduce the rate of SSI by a factor of 3 (6% vs 19%), enhance the return of intestinal motility (5.6 vs 6.5 d), and reduce hospital stay by 20%.^[37]^ However, the SSIs were similar among the 3 groups in our trial. The reason may be attributed to the ERAS guidelines adopted in our hospital. Mild hypothermia can also affect drug metabolism and pharmacokinetics and prolong recovery time, which is consistent with our conclusions.^[38]^ Though a previous study stated that laparoscopic surgery could reduce the radiation heat loss by avoiding abdominal incision, the peritoneal surface is exposed to a large volume of CO_2_ gas during pneumoperitoneum.^[39]^ Previous multivariate logistic regression analysis revealed that anesthesia time and volume of CO_2_ were the only independent risk factors for perioperative hypothermia during laparoscopic colorectal surgery.^[40]^ However, both these factors are similar in our trial. Considering the cost-effectiveness analysis of perioperative warming, current guidance highlights the need to carry out prophylactic measures to avoid unintentional perioperative hypothermia during laparoscopic colorectal surgery.^[24]^ The higher additional costs of the humidification system cannot be weighed against its benefits; as a result, forced-air warming devices can be used as a routine application to maintain normothermia during abdominal surgery. In 2002, the European Association for Endoscopic Surgery recommended the use of warmed irrigation and external warming devices instead of heated insufflation systems to maintain perioperative normothermia for both economic reasons and previous experimental conclusions that stated no clinical benefits of warmed humidified insufflation gas.^[41]^ However, the majority of participants in that study were 30 to 60-year-old women. There are no studies that included participants aged greater than 65 years. Therefore, we only recruited elderly patients between 65 and 75 years in this trial.

Although there is thus far no definite conclusion about whether PetCO_2_ is reliable in predicting partial pressure of carbon dioxide (PaCO_2_) in elderly patients undergoing laparoscopic surgery with CO_2_ inflation, a previous study has confirmed the reliability of PetCO_2_ monitoring in predicting PaCO_2_ in ASA grades 1 and 2 participants.^[42]^ We adopted mainstream end-tidal CO_2_ measurement in our study because of the more accurate estimation of arterial CO_2_ compared with sidestream measurement. Pneumoperitoneum could increase both mean blood pressure and heart rate because of catecholamine release and resistance in the systemic vessels, However, intraoperative normothermia can alleviate this reaction.^[43]^ Consistent with this previous study, we also found that both mean blood pressure and heart rate were significantly increased in group CE in our trial.

A previous study stated that POI is an important factor to prolong the length of hospital stay even in the context of ERAS-guidelines compliant laparoscopic colorectal surgery.^[44]^ We did not find any difference among the 3 groups with respect to POI. The reason for this inconsistency may be due to the relatively small intraoperative temperature change in our trial. Besides, meta analyses have reported continuous IV lidocaine infusion could reduce the interval to return of bowel function and duration of hospital stay. The mechanism is varied, including anti-inflammatory effect, specific inhibition of intestinal sympathetic plexuses, reduction of sympathetic activity, and morphine-sparing effect.^[45–47]^ SSIs represent up to 20% of all health care-associated infections and have been recognized as an important factor of prolonging the hospitalization time of patients especially for colorectal cancer surgery. Several mechanisms have been involved such as subcutaneous vasoconstriction, detrimental effect on the host's ability to mount an immune response, and reduction of the extent of peritoneal dessication.^[48,49]^ Although the discharge time was longer in group CE, we did not record any difference among the 3 groups with respect to SSIs.

We recorded a significant increase of prothrombin time, activated partial thromboplastin time, and thrombin time in group CE in this trial, likely because of hypothermia. However, we did not find any differences between groups WH and CF with respect to coagulation function though both mean skin temperature and body temperature were higher in group CF from the beginning of pneumoperitoneum to 20 minutes after the pneumoperitoneum. The reason may be because coagulation dysfunction is solely concerned with core hypothermia. Previous studies found that hypothermia can affect the function of platelet membrane receptors and reduce the number of circulating blood platelets and the expression of platelet surface membrane glycoprotein, and then inhibit platelet adhesion and aggregation.^[50]^ Hypothermia may also decrease the concentrations of various coagulation factors and fibrinogen, inhibit the coagulation cascade, and ultimately lead to coagulation dysfunction.^[51,52]^

There are some limitations to our study. First, all patients went through preoxygenation with 100% oxygen before induction of anesthesia. However, preoxygenation with 100% oxygen would result in atelectasis. Although we expanded the lungs after induction to airway pressure of 3.92 kPa according to a published literature, it cannot completely eliminate the atelectasis which may have influenced the accuracy of PetCO_2_.^[53]^ Second, though previous studies found that men showed significantly less variability in temperature change than women, we found no sex-related differences in our study.^[54]^ Third, our study included patients undergoing several types of surgery, thereby necessitating different positions. Finally, this trial only investigated the short-term clinical effects of warm, humidified CO_2_ insufflation. Its long-term benefits of peritoneal fibrinolysis and adhesion formation merits further study.

In conclusion, use of either warm, humidified insufflations of CO_2_ or 20°C, 0% relative humidity CO_2_ combined with forced-air warmer set to 38°C during insufflations can both reduce intraoperative hypothermia, coagulation dysfunction, early postoperative cough pain, sufentanil consumption, days to first flatus and solid food intake, and length of hospital stay.

## Author contributions

**Conceptualization:** Rongjuan Jiang, Yan Sun, Huaiming Wang, Min Liang, Xianfeng Xie.

**Data curation:** Rongjuan Jiang, Huaiming Wang, Min Liang.

**Formal analysis:** Yan Sun.

**Methodology:** Yan Sun.

**Visualization:** Rongjuan Jiang.

**Writing – original draft:** Yan Sun, Huaiming Wang, Min Liang, Xianfeng Xie.

**Writing – review & editing:** Xianfeng Xie.
